# Assessment of dietary intake among pregnant women in a rural area of western China

**DOI:** 10.1186/1471-2458-9-222

**Published:** 2009-07-09

**Authors:** Yue Cheng, Michael J Dibley, Xueli Zhang, Lingxia Zeng, Hong Yan

**Affiliations:** 1 Department of Public Health, Xi'an Jiaotong University College of Medicine, Xi'an, Shaanxi 710061, PR China; 2 School of Public Health, University of Sydney, Room 307A, Edward Ford Building (A27), University of Sydney, NSW 2006, Australia; 3 George Institute for International Health, PO Box M201, Sydney, NSW 2050, Australia

## Abstract

**Background:**

Adequate maternal nutrient intake during pregnancy is important to ensure satisfactory birth outcomes. There are no data available on the usual dietary intake among pregnant women in rural China. The present study describes and evaluates the dietary intake in a cohort of pregnant women living in two counties of rural Shaanxi, western China.

**Methods:**

1420 pregnant women were recruited from a trial that examined the effects of micronutrient supplementation on birth outcomes. Dietary information was collected at the end of their trimester or after delivery with an interviewed-administrated semi-quantitative food frequency questionnaire (FFQ). Nutrients intake was calculated from the FFQ and compared to the Estimated Average Requirements (EAR). The EAR cut-offs based on the Chinese Nutrition Society Dietary Reference Intakes (DRIs) were used to assess the prevalence of inadequate dietary intakes of energy, protein, calcium, zinc, riboflavin, vitamin C and folate. Mann-Whitney U and Kruskal Wallis tests were used to compare nutrient intakes across subgroups.

**Results:**

The mean nutrient intakes assessed by the FFQ was similar to those reported in the 2002 Chinese National Nutrition and Health Survey from women living in rural areas except for low intakes of protein, fat, iron and zinc. Of the participants, 54% were at risk of inadequate intake of energy. There were high proportions of pregnant women who did not have adequate intakes of folate (97%) and zinc (91%). Using the "probability approach", 64% of subjects had an inadequate consumption of iron.

**Conclusion:**

These results reveal that the majority of pregnant women in these two counties had low intakes of nutrients that are essential for pregnancy such as iron and folate.

**Trial registration:**

ISRCTN08850194.

## Background

Maternal diets during pregnancy need to provide energy and nutrients for the mother as well as for fetal growth [1,2]. Poor maternal nutrition during pregnancy, particularly during the third trimester, is a major cause of low birth weight (LBW) in developing countries [3,4]. LBW is associated with an increased risk of morbidity and mortality [5] and postnatal growth retardation, which may have adverse long-term effects on physical and cognitive development of the infant, such as chronic heart disease and type II diabetes [6]. Inadequate intakes of specific nutrients in pregnancy have been reported to lead to a variety of poor maternal and infant outcomes. Severe protein and energy shortages have been found to result in a drop of birth weight as observed in the Dutch famine of the winter of 1944–1945 [7]. Inadequate intake of iron in pregnancy can lead to maternal anemia [8] and increased risks of maternal mortality if the anemia is severe [9]. Iron deficiency is also associated with increased risks of low birth weight and preterm delivery [8]. Low periconceptional folate intake increases the risks of neural tube defects [10].

The results of the 2002 Chinese National Nutrition and Health Survey (2002 NNHS) revealed a wide range of nutritional status in populations across China from deficiency states to over nutrition, both of which can be potentially be detrimental to health. Compared to earlier national nutrition surveys it appeared that the quality of the diet of the Chinese population as a whole was improving, but there were significant differences between rural and urban areas [11] including differences between pregnant women in these areas [12]. There are few studies that have reported dietary intake in pregnant women from rural China [12,13]. Furthermore many of these studies have methodological limitations such as measurement of dietary intake over short periods of time and no assessment of the adequacy of dietary intake compared to Estimated Average Requirements (EAR). This study aimed to describe nutrient intakes of pregnant women from rural western China during the third trimester using a semi-quantitative food frequency questionnaire (FFQ) and to assess the adequacy of the dietary intake by comparing the results with EAR.

## Methods

This dietary intake assessment study was a sub-study of a double-blind cluster randomized control trial investigating the effect of micronutrient supplementation on birth outcomes. The methods and description of the study population of the supplementation trial have been described in detail elsewhere [14]. But briefly the study sample in the trial consisted of all women resident in the two counties who became pregnant during the study period and who fulfilled trial selection criteria. Between June 2004 and January 2006, 1421 of these women who were participating in the trial regardless of the treatment group, and who were in their third trimester of pregnancy were recruited for the dietary assessment. These pregnant women were residents of Changwu County and Bin County and were less than 28 weeks of gestation at enrolment. Women consuming nutrient supplements for more than two weeks during their pregnancy were excluded. Pregnant women who could not report their dietary intake because of limited cognitive capacity were also excluded from the dietary survey. The two counties in which the study was conducted were categorized in the second lowest level of economic development for rural counties in China (type 3 out of 4 levels of rural counties) [15]. All women signed a consent form before being interviewed using FFQ. The study was approved by the Human Research Ethics Committee of Xi'an Jiaotong University (Number: 2002001).

Socio-demographic information was collected by structured questionnaire at baseline of the supplementation trial. Weight and height were measured at enrolment into the trial and at the two subsequent antenatal care checks, by trained maternal and child health (MCH) staff of local hospitals. Body Mass Index (BMI) was calculated from measured body weight and height (kg/m^2^). Underweight was defined as a BMI less than 18.5 kg/m^2^. A household wealth index was constructed from an inventory of 16 household assets or facilities with a principal component analysis method [16] and this index was ranked and then the households categorized into three equal portions as an indicator for the poorest, middle income, and richest households [14]. A season was assigned to each subject based on the date one and a half months prior to the endpoint of the dietary recall period: March-May were classified as spring; June-August as summer; September-November as autumn; and December-February as winter.

Dietary intake was assessed using a 68-item semi-quantitative FFQ previously validated in 125 pregnant women in two counties of the Shaanxi province, China [17]. The FFQ was administered in the subject's home by trained staff two weeks before the expected delivery date based on the subject's last menstrual period. Subjects were asked to recall the frequency of consuming each food item listed in the FFQ during the three months prior to the interview. Subjects who were not available for interview at the end of their third trimester were interviewed within 12 weeks of delivery and hence required to recall food habits during the last three months of pregnancy. The frequency scale was open-ended and was listed as times per day/week/month/three months or never. Estimates of portion sizes were also recorded as large, medium or small based on photo graphs of example foods.

Daily nutrient intakes were calculated as grams of food multiplied by the amount of each nutrient in the food and the frequency of consumption. Frequency ranges were converted to single numbers by taking the average of the minimum and maximum values in the range and dividing by the unit of time, e.g. day, week or month. The 2002 Chinese Food Composition Tables [18] were used to calculate nutrient intakes, except for phytate, by linking it to the FFQ dataset through unique food codes and a special purpose program to calculate nutrient intake scores. Phytate intake was calculated using previously reported phytate values for foods in China [19]. Molar ratios of phytate to zinc, calcium or iron were calculated as mmoL phytate per day/mmoL zinc, calcium or iron per day. The proportion of subjects with ratios above the suggested critical values was calculated using the following cut offs: phytate: calcium > 0.24, phytate: iron > 1, phytate: zinc > 15, phytate × calcium: zinc > 200 [20]. Women whose dietary energy intake was greater than 5000 kcal or less than 500 kcal were removed from analysis.

The results were adjusted using the "reference man" method [21] to permit comparisons with the dietary intake reported in the 2002 NNHS. The "reference man" was defined as males aged ≥ 18 years, undertaking light physical activity, whose daily reference energy intake was 2400 kcal [22]. Therefore, daily nutrient intakes were adjusted by daily nutrient intake times 2400 kcal divided by the recommended nutrient intakes (RNIs) of energy. According to the Chinese (RNIs) [22], pregnant women aged 19–49 years undertaking light physical activity should consume 2300 kcal/day. The dietary intakes of all nutrients were expressed as mg/day per "reference man" when comparing to the results of non pregnant subjects in the 2002 NNHS.

The EAR is the amount of a nutrient that is estimated to meet the nutrient requirement of half the healthy individuals in a life-stage and sex group [23]. EAR cut-offs were used for all nutrients, except iron, and compared to nutrient intakes derived from the FFQ. Currently, the EAR cut-offs that are based on the Chinese Nutrition Society Dietary Reference Intakes (DRIs) can be used to assess the prevalence of inadequate nutrient intakes of zinc, selenium, vitamin A, vitamin D, thiamine, riboflavin, vitamin C and folate. The RNI for energy is the same as EAR for energy. Increments for EAR are recommended for certain nutrients during pregnancy [22]. For the EAR of protein and calcium which were not available in Chinese DRIs, the British and American EAR and corresponding increments for pregnancy [24,25] were substituted. The prevalence of inadequate intakes of nutrients within the group was estimated by counting the number of individuals in the group with usual intakes below the EAR plus increment where appropriate [23].

The distribution of the requirement for iron in menstruating women is positively skewed, so the "probability approach" was used [23]. Assuming the local diet has low iron bioavailability, and then a dietary iron absorption rate of 5% was assumed [26]. Each individual's iron intake was adjusted by deducting the median amount of iron required for basal losses (i.e. 0.77 mg) [27]. Adjusted iron intake was natural log transformed to improve normality of the distribution but was converted to 0.00001 for zero or negative values before transformation. The "probability approach" was applied using previously reported usual dietary iron intake data of a group of menstruating women [26].

Data was analyzed using the software package STATA SE 9.2 [28]. The numbers in the tables are means for absolute intakes and "reference man" adjusted intakes of energy and other nutrients. The Mann-Whitney U test (for two groups) and the Kruskal Wallis tests (for more than two groups) were used for most nutrients which have non-normal distributions even after log transformation.

## Results

### Population characteristics

One woman has been removed from further data analysis because her dietary energy intake was greater than 5000 kcal. Anthropometric and demographic characteristics of the pregnant women are presented in Table 1. Their mean age at delivery was 25.7 years. Most women had no paid employment outside their home and lived from their family's agricultural activities. More than half of the women had completed nine years of compulsory education.

**Table 1:** Anthropometric and demographic characteristics of pregnant women in rural Shaanxi China 2004

	Mean	SD
Age at delivery (years) *	25.7	4.4
Weight at first antenatal care (kg) †‡	52.7	6.1
Height (cm) ¶	158.9	5.1
Gender of offspring (%)		
Male	57.4	
Female	42.6	
Gravidity (%)		
First	50.9	
Second	32.6	
> = 3 times	16.5	
Maternal education (%)		
Primary school or lower	31.7	
Secondary school or higher	68.3	
Maternal occupation (%)		
Farmer	85.9	
Others	14.1	
Household wealth (%)		
Poorest	25.6	
Middle	40.8	
Richest	33.6	
Household orchard (%)		
Yes	73.8	
No	26.2	
Household poultry (%)		
Yes	24.1	
No	75.9	

* Data missing for 2 women

† Median gestation at first antenatal care check was 13; range was from 4 to 31 weeks

‡ Data missing for 44 women

¶Data missing for 3 women

Among the study participants, half of them were having their first pregnancy. The comparisons of age at delivery, body weight and height at first antenatal care check, education, occupation and household wealth for women participating in the dietary study compared to the remaining women in the trial revealed no important differences. Similarly, no nutritionally important or statistically significant differences were observed among women who had their dietary intake assessed before delivery compared to after delivery.

### Nutrient intakes

Table 2 presents the mean nutrient intakes of the studied women compared to the mean intakes in the 2002 Chinese National Nutrition and Health Survey (2002 NNHS)[12,15,29]. In comparison to the mean absolute nutrient intakes by non-pregnant living in rural areas in the 2002 NNHS, we found pregnant women from these two study counties had lower intakes of protein, fat and micronutrients such as iron and zinc; higher intake of energy, carbohydrate and calcium. Regarding the absolute nutrient intakes by pregnant living in rural areas, our study women had lower intake of energy. The proportion of energy derived from carbohydrate was higher than that in the pregnant women in the 2002 NNHS (67% vs. 60%) while the proportions from protein and fat were lower. Compared to national average intake in the 2002 NNHS after the reference man adjustment, the study subjects consumed lower intake of fat, retinol, niacin, zinc and copper. The median intake of phytate, adjusted by the "reference man" method [21] was slightly lower than the average phytate intake overall population as reported in the 2002 NNHS. The proportion of subjects with ratios above the suggested critical values were 64% for phytate: calcium > 0.24, 100% for phytate: iron > 1, 70% for phytate: zinc > 15, and 35% for phytate × calcium: zinc > 200.

**Table 2:** Mean nutrient intakes consumed by pregnant women in rural Shaanxi China 2004

	Mean* nutrient intake based on FFQ (n = 1420)	Mean intake by women in rural areas (2002 NNHS) (n = 12483)	Mean intake by pregnant women in rural areas (2002 NNHS) (n = 255)	Mean† nutrient intake based on FFQ (reference male†/d) (n = 1420)	Overall mean* intake of 2002 NNHS (reference male†/d) (n = 68962)
Energy (Kcal)	2331.9	2317.6	2396.7	2433.3	2250.5
Protein (g)	63.4	65.3	67.9	66.1	65.9
Fat (g)	57.0	71.9	77.5	59.5	76.3
Carbohydrate (g)	391.2	351.1	356.1	408.2	321.2
Dietary fiber (g)	19.6	12.9	13.9	20.4	12.0
Vitamin A (μgRE) ‡	572.0	453.7	486.4	596.8	469.2
Retinol (μg)	103.0	120.8	144.9	107.5	151.1
Total Vitamin E (mg)	40.1	33.9	45.9	41.9	35.6
Niacin (mg)	12.2	-	-	12.7	14.7
Vitamin C (mg)	106.3	95.6	95.0	111.0	88.4
Thiamine (mg)	1.4	1.1	1.1	1.5	1.0
Riboflavin (mg)	0.9	0.7	0.8	1.0	0.8
Calcium (mg)	453.7	375.0	362.4	473.4	388.8
Phosphorus (mg)	1147.6	-	-	1197.5	978.8
Potassium (mg)	2389.6	-	-	2493.5	1700.1
Magnesium (mg)	407.6	-	-	425.3	308.8
Iron (mg)	23.2	23.7	24.5	24.2	23.2
Zinc (mg)	8.9	11.3	11.7	9.3	11.3
Selenium (μg)	42.7	-	-	44.5	39.9
Copper (mg)	1.9	-	-	2.0	2.2
Manganese (mg)	7.1	-	-	7.5	6.8
Folate (μg)	265.9	-	-	-	-
Phytate (mg)	1334.3	-	-	1392.3	1186.0
Phytate: calcium	0.2	-	-	-	0.2
Phytate: iron	5.5	-	-	-	4.9
Phytate: zinc	16.8	-	-	-	11.1
Phytate × calcium:zinc (mmol/day)	168.2	-	-	-	89.0

* The median daily phytate intake and median molar ratio of phytate to calcium, iron, zinc and phytate × calcium:zinc are presented.

† The reference man is defined as males aged 18 years, undertaking light physical activity, whose daily reference energy intake is 2400 kcal [21]. Therefore, daily nutrient intakes were adjusted by daily nutrient intake times 2400 kcal divided by her recommended nutrient intakes (RNIs) of energy. The equation is expressed as dietary intakes × 2400/2300 (Pregnant women RNI of energy [21]).

‡ Calculated as: 1 μgRE Vitamin A = 1 μg retinol+ 1/6 μg carotene.

Legend: Mean daily nutrient intakes and molar ratios of dietary phytate: calcium, iron and zinc, and phytate × calcium: zinc pregnant women in rural Shaanxi China 2004 compared to the 2002 Chinese National Nutrition and Health Survey (2002 NNHS) [12,15].

Table 3 illustrates the proportions of subjects with inadequate nutrient intakes compared to EAR [22,24,25]. More than half had inadequate energy intake. There were a high proportion of pregnant women who had an inadequate intake of folate (97%) and zinc (91%). Using the "probability approach", we found that 64% of the subjects had an inadequate intake of iron.

**Table 3:** Median intake and percentage of inadequate intake of selected nutrients in rural Shaanxi China 2004

	Median Intake	EAR*	Increment for pregnancy	<EAR + increment (%)
Energy (Kcal)	2234.6	2100†	200	54
Protein (g)	60.6	38‡	12	31
Calcium (mg)	412.9	525¶	0	70
Zinc (mg)	8.5	8.3	5	91
Riboflavin (mg)	0.88	1.0	0.45	91
Vitamin C (mg)	89.4	75	-9	34
Folate (μg)	244.2	320	200	97

* Estimated Average Requirement for non-pregnant women aged above 18 with low physical activity, China Nutrition Society, 2001

† EAR for energy shown in the table is the figure RNI for energy

‡ The figure shown is the EAR for protein for non-pregnant women aged from 19–50 in USA with the addition of an increment for pregnancy (EAR for groups Food and Nutrition Board, Institute of Medicine, National Academies)

¶The figure shown is the EAR for calcium for non-pregnant women aged 19–50 years in United Kingdom with the addition of an increment for pregnancy (EAR; Department of Health, 1991)

**Figure 1:**
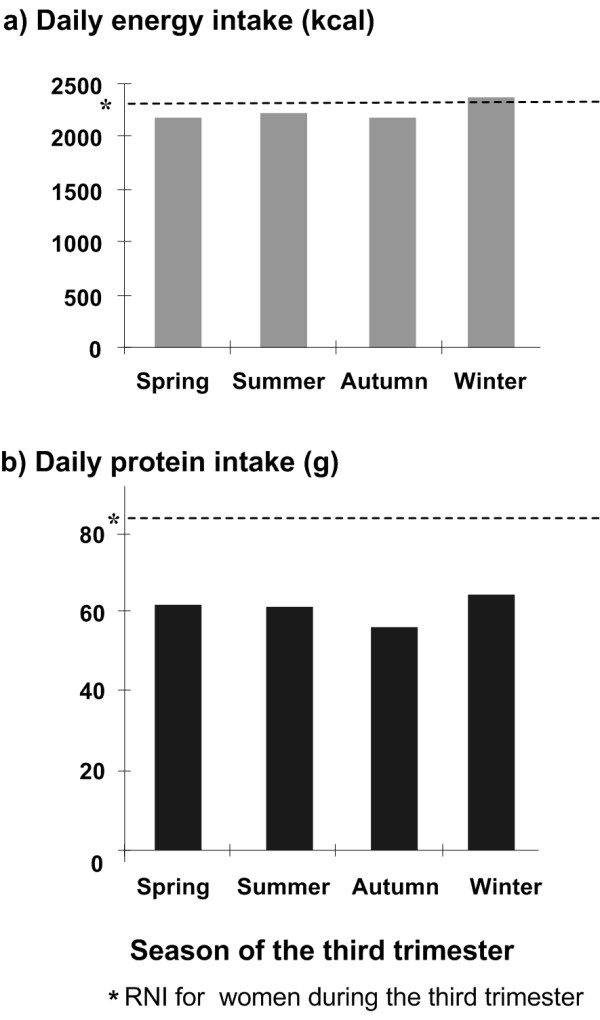
**Comparison of dietary intake of energy and protein across season of third trimester**. Comparison of dietary intakes of energy (kcal) and protein (g) based on the food frequency questionnaire by pregnant women in rural Shaanxi China 2004 by season of third trimester.

**Figure 2:**
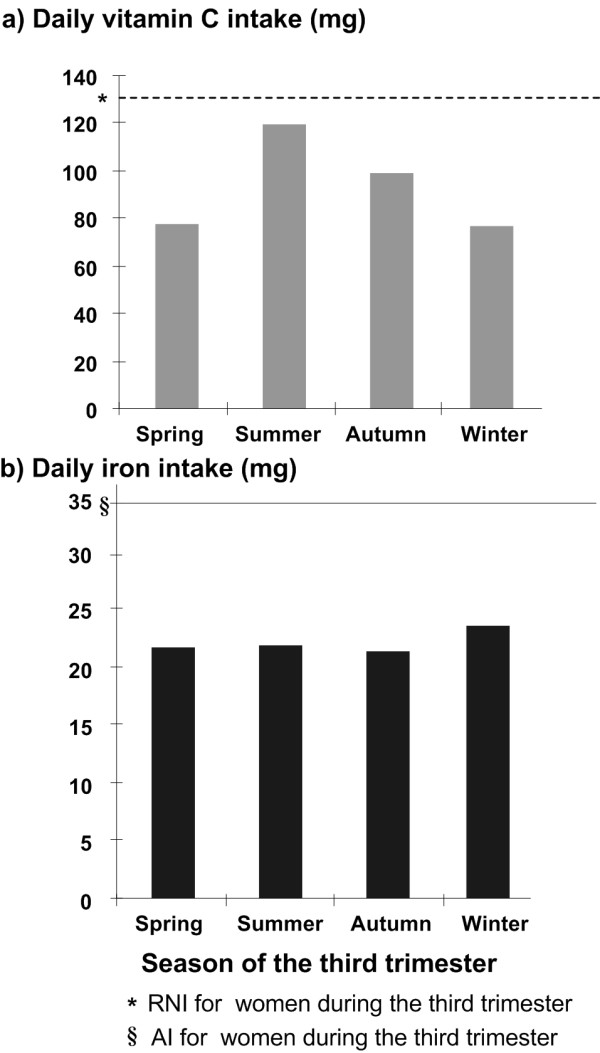
**Comparison of dietary intake of vitamin C and iron across season of third trimester**. Comparison of dietary intakes of vitamin C (mg) and iron (mg) based on the food frequency questionnaire by pregnant women in rural Shaanxi China 2004 by season of third trimester.

The median intakes of energy, protein, calcium, iron, zinc, riboflavin, vitamin C and folate were compared between BMI and household wealth sub-groups (Table 4). Women with a BMI ≥ 18.5 kg/m^2 ^had higher median intakes of energy, protein, iron and zinc compared to women with a BMI < 18.5 kg/m^2 ^(Mann-Whitney U test P < 0.05). Intakes of iron and zinc varied between family household wealth index tertiles (Kruskal-Wallis test P < 0.05). Stratification by season revealed variation of intakes of energy and protein by season (Kruskal-Wallis test P < 0.05, Figure 1). Pregnant women consumed more energy and protein in winter compared to other seasons. Figure 2 demonstrates increased vitamin C intake in summer and autumn (Kruskal-Wallis test P < 0.05). Iron intake was higher in winter but the difference did not reach statistical significance (Kruskal-Wallis test P > 0.05).

**Table 4:** Comparison of selected nutrient intake by characteristics of pregnant women in rural Shaanxi China 2004

		Maternal BMI*			Household wealth			
		
		<18.5 n = 161	> = 18.5 n = 1213	p†	Poorest n = 364	Middle n = 579	Richest n = 477	p‡
Energy (kcal)	Median	2108.7	2250.7	0.015	2200.5	2214.5	2263.1	0.111
	p5, p95¶	1288.0, 3176.4	1305.4, 3644.6		1263.4, 3546.8	1292.8, 3726.5	1366.4, 3603.1	
Protein (g)	Median	55.7	61.3	0.002	59.2	59.7	62.3	0.151
	p5, p95¶	33.0, 91.7	33.5, 105.0		35.2, 101.7	33.1, 103.1	33.5, 105.75	
Calcium (mg)	Median	424.1	416.8	0.539	384.1	398.9	447.4	<0.001
	p5, p95¶	193.5, 781.3	189.3, 856.9		183.9, 825.7	180.6, 849.8	203.1, 867.7	
Iron (mg)	Median	20.6	22.3	0.028	21.6	21.7	22.6	0.016
	p5, p95¶	12.4, 33.8	12.5, 38.6		12.2, 36.3	12.0, 38.4	13.2, 38.2	
Zinc (mg)	Median	7.98	8.60	0.022	8.17	8.50	8.77	0.012
	p5, p95¶	4.59, 12.34	4.81, 14.72		4.62, 13.91	4.62, 14.70	4.90, 14.38	
Riboflavin (mg)	Median	0.84	0.88	0.078	0.82	0.88	0.92	0.002
	p5, p95¶	0.41, 1.42	0.47, 1.59		0.44, 1.55	0.44, 1.60	0.48, 1.59	
Vitamin C (mg)	Median	90.0	89.8	0.715	77.4	88.0	102.1	<0.001
	p5, p95¶	24.4, 220.6	22.2, 246.1		21.2, 228.1	18.6, 242.4	29.4, 260.52	
Folate (μg)	Median	236.0	247.8	0.095	228.2	240.0	268.0	<0.001
	p5, p95¶	104.6, 456.4	114.4, 509.2		116.9, 477.9	104.0, 487.2	126.8, 519.7	

* Data missing for 46 women

† Mann-Whitney U test

‡ Kruskal Wallis test

¶5^th ^percentile – 95^th ^percentile of nutrient intake

## Discussion

Pregnant women in their third trimester in rural Shaanxi, western China, had lower level of dietary intake for most nutrients compared to dietary intake by pregnant women from rural areas reported in the 2002NNHS. Using EAR to evaluate the adequacy of the nutrient intake of the pregnant women from these two counties, we found more than half of the women had inadequate energy intake and the majority of them were at risk of inadequate intake of iron, zinc, riboflavin and folate. In this study the population nutrient intakes were affected by different levels of household wealth index and seasons.

The dietary measurement method (FFQ) was used to assess usual nutrient intake of these study participants. However FFQ might under- or overestimate nutrient intakes depending on the number of food groups included in the FFQ. In our validation study, we observed that the FFQ in general slightly overestimated nutrient intakes. Despite this the FFQ method assesses long term intake and can be applied in large population studies. This method has been increasingly used in epidemiological studies to assess the diets of different populations including pregnant women [30,31] in both developed countries and in China [32,33]. As demonstrated by the validation study, the FFQ had adequate reproducibility and validity for most nutrients. The FFQ had acceptable agreement compared to the mean intakes of six 24-h recalls with only modest over-reporting (FFQ vs. average of six 24 h recalls) for nutrients of 102% to 112%. Nevertheless, the tendency for overestimation of intake by FFQs should be taken into account when interpreting the results of the present study. We may have underestimated the proportion of women who had inadequate intake of nutrients judged by EAR, for example with food energy intake. Although the nutrient intakes were calculated using the 2002 Chinese Food Composition Tables which have been specifically created based on analysis of foods from China there are some limitations to this method. The calculation of nutrient intakes using the food composition tables assumes the nutrient content of foods is constant across China. Although multiple food samples were collected to analyze for nutrient contents, this sampling was not nationwide for all foods [18]. Furthermore, some calculated nutrient intakes, such as selenium, folate, and vitamin C may be less stable and altered by different food preservation, processing and cooking methods used in different parts of China [34]. In the counties where our study was conducted the main sources of foods are locally cultivated and the nutrient content is likely to vary mainly due to losses in storage and cooking.

Our study population consumed more carbohydrate and less energy and fat, compared to the 2002 NNHS that measured nutrient intakes via three days of 24-hour recalls in pregnant women in rural China [12]. It suggests that the pregnant women from these two counties eat more carbohydrate to meet their demand for energy either because of limited sources of protein in their diets, or because of the local dietary preference for wheat products. The study population also had higher intakes of total vitamin A, vitamin C and calcium. Though these higher intakes of nutrients could possibly be explained by the expected over-reporting of nutrient intake as noted in the validation study for the FFQ used. However, the study population consumed less retinol, total vitamin E and zinc, some of which may be explained by the lower intake of meat. This explanation for low intakes of these nutrients has also been proposed in a study of dietary intake of adults in Guangdong Province, China [33].

Phytate is a stored form of phosphorus and is found in high level in unrefined cereal grains, nuts and legumes. It inhibits the absorption of minerals by forming insoluble complexes [35]. Intake of phytate by pregnant women in our study was similar to other rural areas of China and developed countries, but lower than in Africa [20]. Iron, zinc and calcium are essential minerals for which Chinese pregnant women have been reported to have inadequate dietary intake [12]. At the same time, the inhibitory effect of phytate on iron and zinc bioavailability has been reported amongst rural residents in China [20]. Total iron intake in our study was close to the 2002 NNHS values [15], which were often higher than intake of dietary iron reported from developed countries [30,31]. All of the subjects had a phytate: iron molar ratio > 1, indicating that the bioavailability of iron of all subjects was inhibited. Similarly, in rural Guizhou, pregnant women consumed 17.8 mg iron/day, which accounted for 87.6% of RNI, but 22% were diagnosed with anemia [36]. The low bioavailability of iron from the plant-based diets in rural China may explain the high prevalence of anemia in these populations. We found that 64% of the pregnant women surveyed had an inadequate iron intake assessed by the "probability approach" using EAR for menstruating women. This maybe an underestimate as the iron requirement during pregnancy is higher than for menstruating women. Ninety percent of women did not meet the EAR for zinc. Human and animal experiments show that maternal zinc deficiency may cause multiple adverse pregnancy outcomes such as intrauterine growth retardation, teratogenesis, or embryonic or fetal death [2,37] The majority of women surveyed had dietary phytate: zinc molar ratios above the critical level and higher than overall national mean intake in the 2002 NNHS [20]. These results suggest that the pregnant women in these two counties have a higher risk of impaired zinc bioavailability from high phytate intake compared to the overall nation population across China.

The use of the EAR for calcium for British women meant that we did not identify the true prevalence of calcium deficiency. The fact that 80% of the women surveyed did not meet the EAR for calcium of British women implies there was a dietary deficiency of calcium in pregnant women in rural China. Calcium deficiency has been reported as a problem among Chinese residents because of their usual plant-based food patterns [38]. A higher percentage of the women surveyed had diets with phytate: calcium > 0.24 compared to population from rural areas (64% vs.53%) [19] and these findings suggest that the calcium bioavailability among 64% of the study women was affected by phytate.

Table 3 also shows that 54% of women failed to meet the EAR plus recommended pregnancy increment for energy and 31% for protein. Similarly, from a dietary assessment study among pregnant women in Sheffield, UK, a city facing economic difficulties since the decline of the steel industry in the 1970s, 60% of pregnant women of Caucasian ethic origin studied, 60% of the pregnant women did not meet the EAR for energy [30]. In our study, 91% of women did not meet the EAR for riboflavin. This is consistent with the 2002 NNHS data, which reported the main nutrient deficiencies in the Chinese population included riboflavin [39]. Ninety six percent of the subjects failed to meet the EAR for folate, a novel finding that is supported by the demonstration of low plasma folic acid concentrations in rural China [40]. Folate deficiency during pregnancy is also widely reported in developed countries [41]. We found that 34% of our subjects failed to meet the EAR for vitamin C, unlike the relatively high percentage of inadequate intake of other nutrients such as riboflavin, folate. However, because vitamin C is water-soluble and heat labile, recommendations must stress the importance of the retention of vitamin C during food preparation and cooking.

Pregnant women with a BMI ≥ 18.5 kg/m^2 ^consumed significantly more energy and protein than women with a BMI < 18.5 kg/m^2 ^demonstrating the association between body size and demand for macronutrients. At the same time, there were no significant differences in energy and protein intakes found between subgroups of household wealth. Similarly, an English study, conducted in Sheffield, a city with 0.531 million population confronting economic difficulty, showed no differences in nutrient intakes among Caucasian pregnant women according to socio-economic status [30]. However, significant differences were found in calcium, iron, zinc, riboflavin and vitamin C intakes across the various subgroups of household wealth. Study in Austria [42] also found the dietary intake of these nutrients, which are less correlated with energy, varied between different socio-economic status groups. These results indicate the disadvantaged pregnant women are more likely to have micronutrient deficiencies. We found seasonal variations in the intake of energy, protein, calcium, riboflavin and vitamin C by pregnant women. Seasonal fluctuations have commonly been reported for vegetable and fruits intake in Northern rural China [43], and similar fluctuations in these foods would be expected in rural Shaanxi province.

All nutrient intakes reported here were dietary intakes without considering the nutrient supplementation given in the trial. These supplements were not thought to be likely to influence the food intake of the participants in this study.

## Conclusion

Pregnant women in their third trimester in rural Shaanxi, western China, had low dietary intake for most nutrients, especially for nutrients crucial in pregnancy such as iron, zinc, riboflavin and folate. This study highlights the need to develop programs to improve the dietary intake of pregnant women from disadvantaged communities in western China to optimize their health and that of their infants.

## Competing interests

The authors declare that they have no competing interests.

## Authors' contributions

YC designed the study, coordinated the data collection, analyzed and interpreted the data and prepared the manuscript. MD contributed to the study design, helped to analyze and interpret the data and draft the manuscript. XZ helped to analyze and interpret the data and draft the paper. LZ participated in its design and coordination of filed work and data collection and helped to draft the manuscript. HY was the principal investigator; contributed to conceptualizing the study, supervision the field operation, edited the paper, and is guarantor. All authors read and approved the final manuscript.

## Pre-publication history

The pre-publication history for this paper can be accessed here:


